# Global gene expression analysis and regulation of the principal genes expressed in bovine placenta in relation to the transcription factor AP-2 family

**DOI:** 10.1186/1477-7827-5-17

**Published:** 2007-04-27

**Authors:** Koichi Ushizawa, Toru Takahashi, Misa Hosoe, Hiroko Ishiwata, Kanako Kaneyama, Keiichiro Kizaki, Kazuyoshi Hashizume

**Affiliations:** 1 Reproductive Biology Research Unit, Division of Animal Sciences, National Institute of Agrobiological Sciences, 2 Ikenodai, Tsukuba, Ibaraki 305-8602, Japan; 2 Department of Technology, National Livestock Breeding Center, 1 Odakurahara, Odakura, Nishigo, Fukushima 961-8511, Japan; 3 Department of Veterinary Medicine, Faculty of Agriculture, Iwate University, 3-18-8 Ueda, Morioka, Iwate 020-8550, Japan

## Abstract

**Background:**

Cell-cell communication is an important factor in feto-maternal units during placentogenesis. The placenta produces pivotal hormones and cytokines for communication between cotyledonary villi and the maternal caruncle. Gene expression in bovine placenta throughout pregnancy was comprehensively screened by a cDNA microarray, and we searched for a common transcription factor in a gene cluster that showed increasing expression throughout gestation in cotyledonary villi and caruncle.

**Methods:**

Placentomal tissues (villi and caruncle) were collected from Day 25 to Day 250 of gestation for microarray analysis. Global gene expression profiles were analyzed using the k-means clustering method. A consensus sequence cis-element that may control up-regulated genes in a characteristic cluster was examined in silico. The quantitative expression and localization of a specific transcription factor were investigated in each tissue using quantitative real-time RT-PCR and in situ hybridization.

**Results:**

The microarray expression profiles were classified into ten clusters. The genes with most markedly increased expression became concentrated in cluster 2 as gestation proceeded. Cluster 2 included placental lactogen (CSH1), pregnancy-associated glycoprotein-1 (PAG1), and sulfotransferase family 1E estrogen-preferring member 1 (SULT1E1), which were mainly detected in giant trophoblast binucleate cells (BNC). Consensus sequence analysis identified transcription factor AP-2 binding sites in some genes in this cluster. Quantitative real-time RT-PCR analysis confirmed that high level expression of transcription factor AP-2 alpha (TFAP2A) was common to cluster 2 genes during gestation. In contrast, the expression level of another AP-2 family gene, transcription factor AP-2 beta (TFAP2B), was extremely low over the same period. Another gene of the family, transcription factor AP-2 gamma (TFAP2C), was expressed at medium level compared with TFAP2A and TFAP2B. In situ hybridization showed that TFAP2A, TFAP2B and TFAP2C mRNAs were localized in trophoblast cells but were expressed by different cells. TFAP2A was expressed in cotyledonary epithelial cells including BNC, TFAP2B was specifically expressed in BNC, and TFAP2C in mononucleate cells.

**Conclusion:**

We detected gestational-stage-specific gene expression profiles in bovine placentomes using a combination of microarray and in silico analysis. In silico analysis indicated that the AP-2 family may be a consensus regulator for the gene cluster that characteristically appears in bovine placenta as gestation progresses. In particular, TFAP2A and TFAP2B may be involved in regulating binucleate cell-specific genes such as CSH1, some PAG or SULT1E1. These results suggest that the AP-2 family is a specific transcription factor for clusters of crucial placental genes. This is the first evidence that TFAP2A may regulate the differentiation and specific functions of BNC in bovine placenta.

## Background

The placenta that connects the mother to the fetus plays a crucial role in mammalian fetal growth and maintenance of the pregnancy. The mechanisms of implantation, placentation, fetogenesis and delivery are unclear because the complicated cell-cell communication involved is modulated by hormones, cytokines and growth factors. At each stage in gestation, intricate molecular and biochemical regulation is involved in maintaining the fetal-maternal relationship. Placentomes consisting of fetal and maternal tissues, namely cotyledons and caruncles, develop step-by-step during gestation in cattle [1]. The giant trophoblast binucleate cells (BNC) characteristically appear early in gestation and represent approximately 20% of trophoblast cells throughout gestation in the bovine placenta [2]. BNCs participate directly in modifying the endometrial epithelium, beginning at implantation and continuing until term, and play a major role in feto-maternal communication in ruminants [1]. Although BNCs are known to produce various specific molecules – prolactin-like hormones, pregnancy-associated glycoproteins (PAG), steroid hormones and prostanoids, thus acting as endocrine cells [1,3] – the regulatory mechanisms common to the expression of these molecules remain to be investigated. Analyses of global gene expression profiling reveal a new aspect of the intricate molecular mechanisms in the bovine placenta. Even with new technology, analysis of enormous amounts of genetic information reveals a highly complex situation. We have examined the following gene expression profiles: (i) global gene expression in the placenta, mainly in the caruncle or endometrium in early pregnancy, in order to investigate the genes involved in placentation [4]; (ii) global gene expression in the embryo and extra-embryonic membranes during the implantation period [5]; and (iii) trophoblast cell-specific gene expression in a bovine trophoblast cell line (BT-1) [6] using a custom-made cDNA microarray. Other groups have also studied global gene expression in ruminants using cDNA arrays during the pre- or peri-implantation period, specifically in the 8-cell bovine embryo [7], gastrulation [8], implantation [9] and endometrium [10-12]. Microarray analysis gives information about thousands or tens of thousands of genes simultaneously and suggests biological pathways in organs and cells. However, it is difficult to establish correlations among genes within one gene cluster; gene expression data tend to fluctuate because there is still insufficient information about the bovine genome. A common transcription factor may be utilized for genes in identical clusters with very similar expression patterns [13]. Currently, bioinformatics methods allow genome-wide expression of transcriptional regulatory elements to be analyzed rapidly in humans and/or yeast [14-19]. It would be interesting to identify a common response regulator of the principal genes in the placenta; this could elucidate the mechanism of placentation and the properties of BNC. Here, we investigated global gene expression within the placenta from the initial to the late stages of pregnancy, in order to identify the genes related to placentation and placental maintenance. After microarray analysis, a possible common response regulator for trophoblast-cell functions and the maintenance of gestation was examined by *in silico *analysis, using information about the bovine genome, quantitative real-time RT-PCR (QPCR) and *in situ *hybridization.

## Methods

### Animals and tissue collection

Placentomal tissues for mRNA expression were collected from Japanese Black cows. The necessary extra-embryonic membranes, placenta and endometrium were collected at a local slaughterhouse on days 25 to 28, 56 to 64, 144 to 149 and 245 to 252 after artificial insemination (Day 0) and on Day 13 of the estrus cycle (non-pregnant). The tissues were separated into two portions, the cotyledonary villous (COT) and the caruncle areas (CAR), the latter including the maternal placentomal septa in the endometrium. It was difficult to separate the COT from the fetal membranes on days 25 to 28, as the extra-embryonic membrane (EEM) contained very few villi. Tissue taken from three different cows on days 25, 27 and 28 of gestation (n = 2 animals for the microarray; n = 3 animals for QPCR) was designated Day 25 EEM; Day 25 endometrium was designated Day 25 ENDO. Placentomal tissues were collected on days 56 (two animals), 64 and 65 (totally n = 3 animals for the microarray; n = 4 animals for QPCR) and were designated Day 60 COT and Day 60 CAR. Sample materials from days 144, 148 and 149 (n = 2 animals for the microarray; n = 3 animals for QPCR) and days 245 (two animals), 249 and 252 (totally n = 2 animals for the microarray; n = 4 animals for QPCR) were respectively marked Day 150 COT, Day 150 CAR, Day 250 COT and Day 250 CAR. The cotyledonary and caruncular parts were mechanically separated, with each part containing some of the tissue. Two samples from non-pregnant cows were collected for the microarray. The collected samples were stored at -80°C prior to RNA extraction, and additional placentomes from Day 56 were fixed in 3.7% formaldehyde PBS at pH 7.4 and then embedded in paraffin wax and stored at 4°C prior to *in situ *hybridization. All procedures for these animal experiments were carried out in accordance with guidelines approved by the Animal Ethics Committee of the National Institute of Agrobiological Sciences for the use of animals.

### Sample RNA preparation

Total RNA was individually isolated from ENDO, CAR, EEM and COT using ISOGEN (NipponGene, Toyama, Japan) according to the manufacturer's instructions. Poly (A)^+ ^RNA was prepared from the total RNA using an Oligotex-dT30 Super mRNA isolation kit (JSR, Tokyo, Japan). The extracted poly (A)^+ ^RNA was used for the cDNA microarray experiment.

### Microarray analysis

#### cDNA microarray

A custom-made utero-placental cDNA microarray developed in our laboratory [4,20] was used. A total of 3955 clones were spotted on one chip; 1780 individual genes were annotated by BLASTn. The details of the cDNA microarray experiments were described in previous reports [4,5].

#### Microarray hybridization

cDNA microarray hybridization was performed as described previously [4,5]. Poly(A)^+ ^RNA was reverse-transcribed with Cy3 or Cy5 fluorescent dye (Amersham Biosciences, Buckinghamshire, UK) using SuperScript II reverse transcriptase (Invitrogen, Carlsbad, CA, USA) to make the hybridization probes. The labeled probes were concentrated in a Microcon filter (Millipore, Bendford, MA, USA), diluted in hybridization solution (a mixture of SSC, SDS, poly(A) and yeast tRNA), and applied to the microarray. After incubation at 65°C, the array chips were sequentially washed with SSC/SDS solution and SSC solution. The hybridization images were scanned using a GenePix 4000B (Axon Instrument, Union City, CA, USA) and analyzed by the GenePix Pro 4.0 program.

Sample hybridizations were performed in duplicate for all samples. The COT and CAR samples from the pregnant cows were reverse-labeled. In the reverse labeling procedure, for example, the cDNAs for COT of Day 60 and CAR of Day 60, which had initially been labeled with the fluorescent dyes Cy3 and Cy5, respectively, were then labeled with Cy5 and Cy3, respectively. The two endometrial samples were self-labeled; for example, the ENDO cDNA samples were labeled with Cy3 or Cy5, respectively, and both labeled cDNA were mixed and hybridized on the microarray. Each data point was individually normalized and the average value was used for data analysis.

#### Data normalization for microarray

Data were normalized by the following procedures [5,21]. The local background intensity of each spot was smoothed by a local weight regression (lowess) smoother and subtracted from the feature intensity data. The subtracted intensity data were subjected to non-parametric regression and local variance normalization. Non-parametric regression can reduce intensity-dependent biases. Compared with linear regression, the accuracy is improved as long as the points in the scatter plot of Cy3 vs. Cy5 are not distributed around a straight line. Normalization of local variance controlled most of the background in low-intensity data, whereas the normalized data, in many cases, showed no significant fold-differences in comparison with the background-subtracted raw intensity ratios, which frequently indicated higher fold-differences. Thus, the variance method employing bovine utero-placental array data produced highly reliable normalized ratios. Compliance with Minimum Information About a Microarray Experiment (MIAME) [22] was assured by depositing all the data in the Gene Expression Omnibus (GEO) repository [23]. The GEO accession numbers are as follows. Platform: GPL1221; Samples: GSM170629, GSM170632, GSM170636, GSM170637, GSM170638, GSM170639, GSM170640, GSM170641, GSM170642, GSM170643, GSM170644, GSM170645, GSM170655, GSM170679, GSM170687, GSM170688, GSM170689, and GSM170690; Series: GSE7096.

#### Cluster analysis of microarray data

The data for individual genes were obtained by averaging the corresponding spots on the microarray. The transformed log_2 _values were used for cluster analysis. The TIGR MultiExperiment Viewer 3.0 (MeV 3.0) program was used for *k*-means cluster analysis [24,25]. The general expression patterns of the 1446 unique genes, except for unreliable low-expression genes, were investigated using the *k*-means algorithm. The data for each gene were represented by an eight-dimensional vector. *K*-means clustering was performed by partitioning around 10 centroids. The distances between the gene vectors were calculated using the cosine coefficient (vector angle).

### The search for a transcription factor common to a cluster

We searched for a transcription factor consensus binding site common to all genes in cluster 2, because the microarray analysis revealed the most marked changes in this cluster. We obtained a region 200 bp upstream from each gene from Map Viewer on the NCBI web site [26]. We searched for a transcription factor binding site common to these upstream regions using the TFBIND program [27,28].

### Quantitative real-time RT-PCR (QPCR)

We investigated the mRNA expression patterns of (i) six characteristic genes selected from the microarray analysis (Annexin I (*ANXA1*), RNA polymerase II carboxy-terminal domain small phosphatase 2 (*CTDSP2*), Msh homeo box 1 (*MSX1*), Heat shock 70 kDa protein 1A (*HSPA1A*), Heat shock 70 kDa protein 8 (*HSPA8*) and Sulfotransferase family 1E estrogen-preferring member 1 (*SULT1E1*)), and (ii) transcription factors for which consensus binding sites were present in multiple members of cluster 2, namely transcription factors AP-2 alpha (*TFAP2A*), AP-2 beta (*TFAP2B*) and AP-2 gamma (*TFAP2C*).

Real-time RT-PCR was performed using the SYBR Green Detection System (Applied Biosystems, Foster City, CA, USA). Fifty nanograms of total RNA was reverse-transcribed for 30 min at 48°C by MultiScribe™ reverse transcriptase with a random primer, dNTP mixture, MgCl_2 _and RNase inhibitor. After heat inactivation of the reverse transcriptase for 5 min at 95°C, PCR and the resulting relative increase in reporter fluorescent dye emission were monitored in real time using an Mx3000P QPCR system (Stratagene, La Jolla, CA, USA). The primer pair was designed by the Primer Express Program (Applied Biosystems). The primers for each gene are listed in Table 1. The thermal cycling conditions included one cycle at 50°C for 2 min, one cycle at 95°C for 10 min, and 40 cycles at 95°C for 15 s and 60°C for 1 min. The relative difference in the initial amount of each mRNA species (or cDNA) was determined by comparing the C_T _values. To quantify the mRNA concentrations, standard curves for each gene were generated by serial dilution of the plasmid containing its cDNA. We confirmed the melting curve for detecting the SYBR Green-based objective amplicon because SYBR Green also detects double-stranded DNA including primer dimers, contaminating DNA and PCR products from misannealed primers. Contaminating DNA or primer dimers appear as a peak separate from the desired amplicon peak. The expression ratio of each gene to *GAPDH *mRNA was calculated to adjust for variations in the RT-PCR reaction. All values are presented as mean ± SEM. QPCR was duplicated on one animal sample. To be more precise: for the Day25 and Day150 samples, QPCR data were collected from 3 animals (biological replicates) and the technique was repeated for one animal sample (technical duplicate); in total, six data were obtained. For QPCR data from the Day60 and Day250 samples, 4 biological replicates were obtained and technical duplicate was performed on one sample (eight data in total). One-way ANOVA followed by the Tukey-Kramer multiple comparison test was used for statistical analysis. Differences were considered significant at *P *< 0.05.

**Table 1:** Oligonucleotide primers used for quantitative real-time RT-PCR analysis

Gene	Primer	Sequence	Position
*ANXA1*	Forward	5' GGCTTTGCTTTCTCTTGCTAAGG 3'	611–633
(NM_175784)	Reverse	5' TGAATCAGCCAAGTCGTCATTT 3'	680–669
*CTDSP2*	Forward	5' GGCCTGGTGTCCAAGTCCT 3'	203–221
(DT808814)	Reverse	5' CAGAAAAGGGCCTTGAAGATGT 3'	267–246
*MSX1*	Forward	5' TCCCTTGTTCAGCACCGC 3'	1207–1224
(NM_174798)	Reverse	5' CGGAGGACAAACCAGAGCA 3'	1270–1252
*HSPA1A*	Forward	5' GCAGACCCGCTATCTCCAAG 3'	41–60
(NM_174550)	Reverse	5' ACCTGAAAACGGCCCACAG 3'	117–99
*HSPA8*	Forward	5' CAAGCTATGTCGCCTTTACTGA 3'	115–136
(NM_174345)	Reverse	5' GGATTCATTGCGACTTGGTTC 3'	188–168
*SULT1E1*	Forward	5' CAGGATCATCTGGACAGTGTACCA 3'	182–205
(NM_177488)	Reverse	5' CCAAGTTTGCCAAAGTAATCTGAA 3'	259–236
*TFAP2A*	Forward	5' CCCAACGAAGTCTTCTGTTCAGT 3'	775–797
(XM_875452)	Reverse	5' ACCTTGTACTTCGAGGTGGAGC 3'	842–821
*TFAP2B*	Forward	5' CGAATGCCTCAATGCGTCT 3'	1090–1108
(BC120374)	Reverse	5' CCCATTTTTCGATTTGGCTC 3'	1150–1131
*TFAP2C*	Forward	5' GGTGTTCTCAGAAGAGCCAAGTC 3'	1016–1038
(BC120401)	Reverse	5' GACATAGGCAAAGTCCCGAGC 3'	1186–1166
*GAPDH*	Forward	5' AAGGCCATCACCATCTTCCA 3'	178–197
(U85042)	Reverse	5' CCACTACATACTCAGCACCAGCAT 3'	253–230

### *In situ *hybridization

Approximately 500 bp cDNA of a representative cluster 2 gene, *SULT1E1*, and of the genes commonly utilized in the cluster, *TFAP2A*, *TFAP2B *and *TFAP2C*, was used as template for synthesizing a hybridization probe. Digoxigenin (DIG)-labeled anti-sense and sense cRNA probes were prepared as described in previous studies [29-31]. Day 56 bovine placentomes were sectioned into 7 μm-thick sections. *In situ *hybridization was performed using an automated Ventana HX System Discovery with a RiboMapKit and a BlueMapKit (Ventana, Tucson, AZ, USA) [29-31]. Briefly, the sections were hybridized with DIG-labeled probes in RiboHybe (Ventana) hybridization solution at 63°C (*SULT1E1 *and *TFAP2A*) or 61°C (*TFAP2B *and *TFAP2C*) for 6 hours, then washed for 3 × 6 min in RiboWash (Ventana) at 65°C and fixed in RiboFix (Ventana) at 37°C, 10 min. The *SULT1E1 *and *TFAP2A *hybridization signals were detected using a monoclonal-anti-digoxin biotin conjugate (Sigma, Saint Louis, MI, USA). The *TFAP2B *and *TFAP2C *hybridization signals were detected with a rabbit polyclonal anti-digoxin HRP conjugate (Dako Cytomation, Carpinteria, CA, USA) using an AmpMapKit (Ventana). After preparation, the hybridized slides were observed with a Leica DMRE HC microscope (Leica Microsystems, Wetzlar, Germany) and a Fujix digital camera HC2500 (Fujifilm, Tokyo Japan).

## Results

### Correlations in the microarray data

We examined the correlations of microarray data among tissues at corresponding stages of pregnancy (Table 2). The correlations for non-pregnant (n = 2 animals), Day25 (n = 2 animals), Day60 (n = 3 animals), Day150 (n = 2 animals) and Day250 (n = 2 animals) samples were calculated in the biological replicates and technical duplicates by reverse labeling. The correlation coefficients between the two sets of Day 25 and the three sets of Day 60 data are high. This is also true for the two sets of Day 150, Day 250 and non-pregnant data. Therefore we used the averages of the two data sets for each of these stages of gestation.

**Table 2:** The correlation coefficients (r) between the same stage of microarray data.

Gestation Days	EEM-COT/r value	ENDO-CAR/r value
Non-Pregnant	---	≥ 0.87
Day25	≥ 0.87	≥ 0.91
Day60	≥ 0.85	≥ 0.86
Day150	≥ 0.85	≥ 0.92
Day250	≥ 0.81	≥ 0.70

The correlations of non-pregnant, Day25, Day150, and Day250 samples were calculated at biological duplicate n = 2 and technical duplicate by reverse labeling n = 2. The correlations of Day60 samples were calculated at biological duplicate n = 3 and technical duplicate by reverse labeling n = 3. Differences were considered significant at *P *< 0.05.

### Cluster analysis of global gene expression in bovine placenta

#### General gene expression

**Figure 1:**
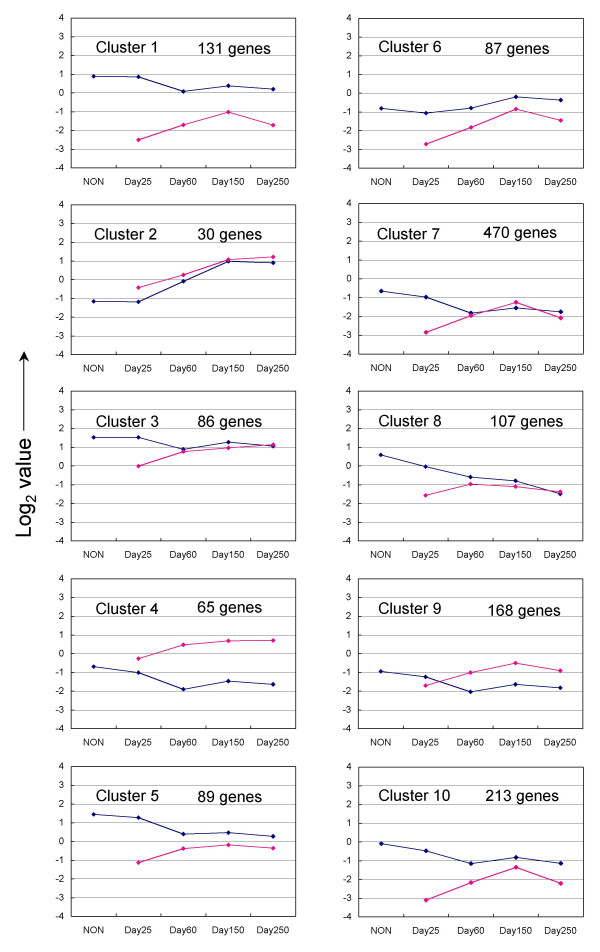
***K*-means clusters of the gene expression pattern from non-pregnant ENDO to Day 250 CAR and Day 25 EEM to Day 250 COT**. The 1446 unique genes except for the genes that exhibited low expression intensity were subjected to clustering analysis. The blue line shows to the *k*-means center of gene expression on ENDO to CAR. The pink line shows to the *k*-means center of gene expression on EEM to COT. The expression intensity refers log2 value of normalized data.

Three hundred and twenty genes out of a total of 1780 were excluded from cluster analysis because of their low expression values. The remaining 1446 genes were partitioned into ten categories by *k*-means clustering, as depicted in Fig. 1. The ten *k*-means cluster profiles were classified into three types: (i) profiles with COT gene-expression intensities higher than those of CAR from Day 60 to Day 250 of gestation (clusters 4 and 9); (ii) those with expression intensities in COT level similar to those of CAR in clusters 2, 3, 7 and 8; and (iii) those with CAR expression intensities higher than those of COT (clusters 1, 5, 6 and 10). The number of genes in each cluster ranged from about 500 to 30. Specifically, cluster 7 contained 470 genes, whereas cluster 2 contained only 30 genes.

#### Specific genes and their expression patterns in the clusters

Cluster 1 comprised 131 genes including numerous ribosomal proteins, osteonectin (*SPARC*), decorin (*DCN*), cytochrome-c oxidase subunit V and Rho GDP dissociation inhibitor beta (*ARHGDIB*). The expression intensities were high in the ENDO-CAR tissues in non-pregnant subjects and declined slightly until Day 60, after which they remained more or less constant until Day 250. In contrast, the expression level was low in EEM-COT tissues on Day 25 and increased up to Day 150. In cluster 2, the expression intensity increased from Day 25 to Day 150 in both ENDO-CAR and EEM-COT. Cluster 2 comprised only 30 genes including placental lactogen (*CSH1*), prolactin-related proteins (*PRPs*), *PAGs *and *SULT1E1*. In cluster 3, gene expression intensities were high in ENDO-CAR and EEM-COT. Expression decreased slightly from non-pregnant to Day 60 in CAR, but increased markedly in EEM-COT from Day 25 to Day 60 and continued to increase gradually up to Day 250. Cluster 3 included 86 genes, mainly cytoskeleton and cell adhesion genes such as beta-actin (*ACTB*), alpha-tubulin (*TUBA*), tropomyosin 2 (*TPM2*), Villin 2 (*VIL2*, ezrin) and chloride-channel calcium-activated family member 3 (*CLCA3*, Lu-ECAM-1). In cluster 4, the profiles of gene expression intensities in EEM-COT and ENDO-CAR were opposite; the intensities increased slightly from Day 25 to Day 250 in EEM-COT, but declined slightly from non-pregnant ENDO to Day 250 in CAR. This cluster included 65 genes such as alpha-lactalbumin (*LALBA*), aldose reductase (*AKR1B1*), the insulin-like growth factor II (*IGF2*), *HSPA1A*, *HSPA8*, and heat shock 27 kDa protein 1 (*HSPB1*). In cluster 5, the gene expression intensity was higher in ENDO than in EEM. Cluster 5 included 89 genes such as type III and XII collagens, Calbindin 3 (*CALB3*), tissue inhibitor metalloproteinase 2 (*TIMP2*), and trophoblast Kunitz domain protein 5 (*TKDP5*). In cluster 6, the expression intensity was low in both ENDO-CAR and EEM-COT. The intensity increased from Day 25 to Day 150. In contrast, the intensity increased markedly from Day 25 to Day 150 and then declined to Day 250. Cluster 6 included 87 genes such as fibronectin (*FN1*), coronin actin-binding protein 2A (*CORO2A*), profilin 1 (*PFN1*), an inhibitor of metalloproteinase 1 (*TIMP1*), the insulin-like growth factor binding protein-3 (*IGFBP3*), the macrophage migration inhibitory factor (*MIF*) and heat shock 90 kDa protein (*HSP90*). In cluster 7, expression was low in both ENDO-CAR and EEM-COT. The intensity declined further to Day 60 and then remained steady up to Day 250. In contrast, the expression intensity in EEM-COT increased significantly from Day 25 to Day 150. Cluster 7 comprised the largest number of genes (470), including 11-beta hydroxysteroid dehydrogenase 2 (*HSD11B2*), Annexin II (*ANXA2*), vinculin (*VCL*), alpha E-catenin (*CTNNA1*), mucin (*MUC1*), RNA polymerase II carboxy-terminal domain small phosphatase 2 (*CTDSP2*), Msh homeo box 1 (*MSX1*), vascular endothelial growth factor (*VEGF*) and *VEGFB*. In cluster 8, the initially rather high intensity in ENDO-CAR decreased throughout the examination period. In contrast the initially low expression intensity in EEM-COT showed a slight increase from Day 25 to Day 60, then decreased slightly up to Day 250. Cluster 8 included 108 genes, such as extracellular matrix (ECM) related genes, type I collagen alpha 2 (*COL1A*2), matrix Gla protein (*MGP*), laminin beta 1 (*LAMB1*), clusterin (*CLU*) and uterine milk protein (*UMP*). In cluster 9, low expression intensities were found in ENDO-CAR and EEM-COT. The expression in ENDO-CAR declined to Day 250. In contrast, it increased slightly in EEM-COT from Day 25 to Day 150 and then decreased to Day 250. This cluster included 168 genes such as stanniocalcin (*STC1*), growth hormone receptor (*GHR*), selectin L (*SELL*), glycoprotein-4-beta-galactosyltransferase 2 (*B4GALT1*), annexin I (*ANXA1*) and cathepsin L (*CTSL*). In cluster 10, expression was low in ENDO-CAR, with the intensity decreasing slightly from non-pregnant to Day 250. An extremely low initial intensity was detected in EEM-COT but this increased greatly from Day 25 to Day 150. Cluster 10 included 213 genes such as S100 calcium binding protein A11 (*S100A11*), apolipoprotein D (*APOD*), cytochrome P450 family 11, subfamily A polypeptide 1 (*CYP11A1*) and matrix metalloproteinase 2 (*MMP2*). The 10 genes representative of the individual clusters are listed in Table 3.

**Table 3:** The representative genes which were distributed to each cluster

Accession No.	Gene Name
Cluster 1	
NM_175797	ARHGDIB: Rho GDP dissociation inhibitor beta
NM_174506	BCKDHA: branched chain alpha-keto acid dehydrogenase
NM_001034046	COX5B: Cytochrome c oxidase subunit Vb
NM_173906	DCN: Decorin
NM_001040498	JSP.1: MHC Class I JSP.1
NM_001015556	RPL18: Ribosomal protein L18
NM_001015531	RPS5: Ribosomal protein S5
NM_174464	SPARC: secreted protein, acidic, cysteine-rich
NM_001002885	TMSB4X: Thymosin beta 4, X chromosome
NM_174491	YWHAE: 14-3-3 epsilon
	
Cluster 2	
NM_181007	CSH1: Placental lactogen
AB098803	LOC404051: Similar to thrombin inhibitor
AB098909	SERPINB6: Serpin peptidase inhibitor clade B member 6
NM_174411	PAG1: Pregnancy-associated glycoprotein 1
NM_176616	PAG5: Pregnancy-associated glycoprotein 5
NM_176618	PAG7: Pregnancy-associated glycoprotein 7
NM_174159	PRP1: Prolactin-related protein 1
M27239	PRP2/4: Prolactin-related protein 2/4
NM_177488	SULT1E1: Sulfotransferase family 1E estrogen-preferring member 1
NM_174623	TMSB10: Thymosin, beta 10
	
Cluster 3	
NM_173979.3	ACTB: Actin, beta
NM_181018	CLCA3: Chloride channel, calcium activated, family member 3
NM_174333	GRP58: Glucose regulated protein 58 kD
M83104	LOC515773: Cytochrome b-5 reductase
XM_870635	FTH1: Ferritin heavy polypeptide 1
NM_001038163	MGC133894: Similar to Tubulin alpha-3 chain
NM_174600	SLC1A3: Solute carrier family 1
NM_001010995	TPM2: Tropomyosin 2
M62428	UBC: Polyubiquitin
NM_174217	VIL2: Villin 2
	
Cluster 4	
NM_001012519	AKR1B1: Aldose reductase
NM_174800	CFDP2: craniofacial development protein 2
AF013213	EEF1A1: Eukaryotic translation elongation factor 1 alpha 1
NM_174550	HSPA1A: Heat shock 70 kD protein 1
NM_174345	HSPA8: Heat shock 70 kDa protein 8
NM_001025569	HSPB1: Heat shock 27 kDa protein 1
NM_174087	IGF2: Insulin-like growth factor 2
NM_174378	LALBA: Lactalbumin, alpha
XM_583697	LOC507139: Similar to grancalcin
NM_180999	LYZ: Lysozyme
	
Cluster 5	
NM_181003	AQP4: Aquaporin 4
NM_174257	CALB3: Calbindin 3
AB099882	COL12A1: Collagen, type XII, alpha 1
NM_001034039	COL1A1: Collagen, type I, alpha 1
NM_174770	GPX4: Glutathione peroxidase 4
NM_001033610	KRT8: Keratin 8
XM_588040	LOC510833: Similar to Collagen alpha 1(III)
NM_174459	SEPP1: Selenoprotein P-like protein precursor
NM_174472	TIMP2: Tissue inhibitor of mettaloproteinase 2
AF241780	TKDP5: Trophoblast Kunitz domain protein 5
	
Cluster 6	
NM_001046249	CALM1: Calmodulin 1
NM_001038220	CORO2A: Coronin, actin binding protein, 2A
K00800	FN1: Fibronectin 1
NM_174076	GPX1: Glutathione peroxidase 1
NM_174343	HSD3B: HSD3B protein
NM_174556	IGFBP3: Insulin-like growth factor binding protein 3
XM_614707	LOC534812: Similar to Heat shock protein HSP 90-alpha
NM_001033608	MIF: Macrophage migration inhibitory factor
NM_001015592	PFN1: Profilin 1
NM_174471	TIMP1: Tissue inhibitor of metalloproteinase 1
	
Cluster 7	
NM_174716	ANXA2: Annexin A2
NM_001045935	CTDSP2: CTD small phosphatase 2
NM_174642	HSD11B2: Hydroxysteroid (11-beta) dehydrogenase 2
XM_612863	LOC533452: Similar to Alpha-1 catenin
NM_174798	MSX1: Msh homeo box 1
NM_174115	MUC1: Mucin 1
NM_205775	TKDP4: Trophoblast Kunitz domain protein 4
BE477825	VCL: Vinculin
NM_174216	VEGF: Vascular endothelial growth factor
NM_174487	VEGFB: Vascular endothelial growth factor B
	
Cluster 8	
NM_173902	CLU: Clusterin
NM_174520	COL1A2: Collagen, type I, alpha 2
NM_174029	CST3: Cystatin C
NM_174031	CTSB: Cathepsin B
NM_176612	HMGB1: High-mobility group box 1
NM_174092	IL1A: Interleukin 1, alpha
XM_598260	LAMB1: Laminin, beta 1
NM_174707	MGP: Matrix Gla protein
NM_174030	CTGF: Connective tissue growth factor
NM_174797	UMP: Uterine milk protein
	
Cluster 9	
NM_175784	ANXA1: Annexin I
AF515786	B4GALT1: Glycoprotein-4-beta-galactosyltransferase 2
NM_174032	CTSL: Cathepsin L
NM_176608	GHR: Growth hormone receptor
NM_178319	GRP: Gastrin-releasing peptide
NM_174125	NPPC: Natriuretic peptide precursor C
NM_176621	PAG10: Pregnancy-associated glycoprotein 10
NM_176619	PAG8: Pregnancy-associated glycoprotein 8
NM_174182	SELL: Selectin L
NM_176669	STC1: Stanniocalcin 1
	
Cluster 10	
BC109863	APOD: Apolipoprotein D
NM_176648	CAPZB: Capping protein (actin filament) muscle Z-line, beta
NM_176644	CYP11A1: Cytochrome P450, family 11, subfamily A, polypeptide 1
NM_174363	INHBA: Inhibin, beta A
NM_174100	LDHB: Lactate dehydrogenase B
NM_001034053	LMNA: Lamin A
XM_615304	LOC541253: Similar to Limbic system-associated membrane protein
NM_174745	MMP2: Matrix metalloproteinase 2
NM_174409	OSTF1: Osteoclast stimulating factor 1
BC102667	S100A11: S100 calcium binding protein A11

### QPCR analysis of representative genes

**Figure 2:**
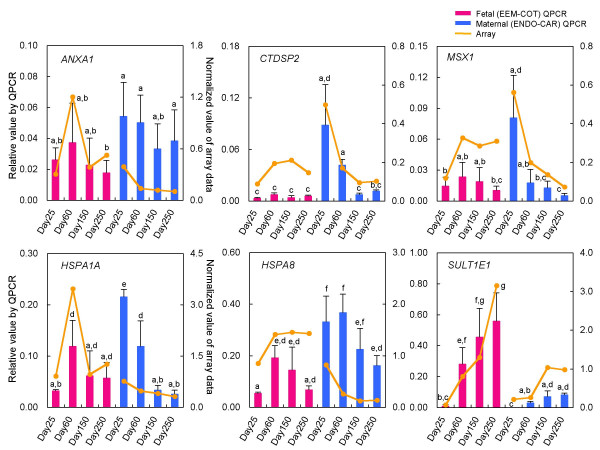
**QPCR analysis and normalized microarray data of *ANXA1*, *CTDSP2*, *MSX1*, *HSPA1A*, *HSPA8*, and *SULT1E1 *mRNA at each stage of bovine tissue (ENDO, CAR, EEM, COT)**. The gene expressions on Days 25 ENDO (n = 3), 60 CAR (n = 4), 150 CAR (n = 3), 250 CAR (n = 4), 25 EEM (n = 3), 60 CAR (n = 4), 150 CAR (n = 3), and 250 CAR (n = 4) are shown. The QPCR expression of these genes was normalized to the expression of *GAPDH *measured in the same RNA preparation. The pink bar shows gene expression on fetal side (EEM to COT) by QPCR. The blue bar shows gene expression on maternal side (ENDO to CAR) by QPCR. The yellow line shows normalized value of the microarray. Values are means ± SEM. Values with different letters are significantly different (*P *< 0.05).

We selected dominant genes for which the expression level was known during the implantation, placentation or embryogenesis stages in other species [32-38]: *ANXA1 *from Cluster 9, *MSX1 *and *CTDSP2 *from Cluster 7, *HSPAs *(*1A and 8*) from Cluster 4 and *SULT1E1 *from Cluster 2. These data are shown in Fig. 2. In CAR, the microarray data for *ANXA1, HSPA1A *and *HSPA8 *were weak relative to the QPCR value. For *CTDSP2, MSX1 *and *SULT1E1*, the QPCR values clearly reflected the microarray data. In general, the QPCR results were consistent with the microarray analysis results.

### The search for a transcription factor common to cluster 2

**Figure 3:**
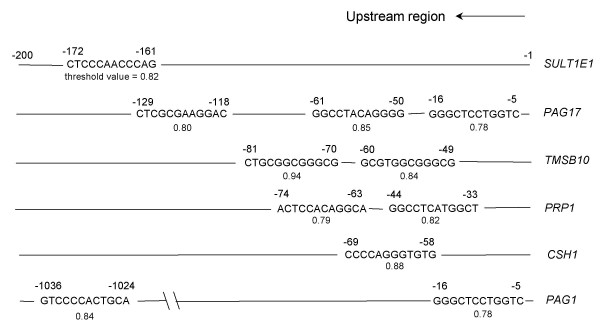
**Potential AP-2 binding site in upstream region (-200 to -1) of principal genes in cluster 2, identified by TFBIND software**. The AP-2 consensus sequence is "MKCCCSCNGGCG" (M = A/C/G; K = A/G/T; S = G/C; N = A/G/C/T) from TRANSFAC databases. The threshold value exhibits homology with the above consensus sequence. "1" represents perfect coincidence with the consensus sequence.

Cluster 2 contained genes with expression intensities that were strong and up-regulated during gestation. This cluster contained many placenta-specific genes such as CSH1, PRPs and PAGs. The transcription factor that commonly regulates these genes is expected to have a pivotal role in the bovine placenta. Some genes were selected from cluster 2 in order to search for the *cis*-element. They were *CSH1*, *PAG1*, *PAG17*, *PRP1*, *SULT1E1 *and thymosin β10 (*TMSB10*), all selected by MapView from the NCBI web site. A transcription factor binding site common to the six upstream region sequences was examined in these genes using TFBIND software [27]. We found that the six sequences had AP-2 binding sites within 200 bp upstream of the transcription start (Fig. 3).

### Localization of cluster 2 genes

**Figure 4:**
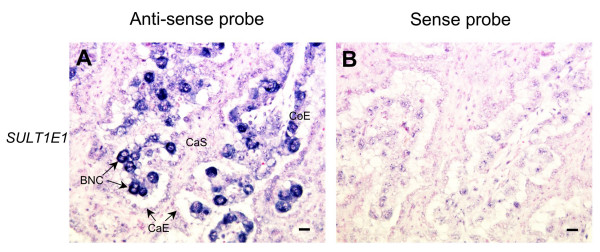
**Localization of *SULT1E1 *mRNA in a bovine placentome on Day 56 of gestation**. *SULT1E1 *mRNA was detected by *in situ *hybridization. (A) DIG-labeled anti-sense cRNA probes were used. (B) DIG-labeled sense cRNA probes were used. Seven-micrometer sections of bovine placentome were hybridized with each probe. Scale bar = 20 μm. CaE: caruncular epithelium. CaS: caruncular stroma. CoE: cotyledonary epithelium. BNC: binucleate cell.

Most of cluster 2-specific genes such as *CSH1, PRP1 *and *PAG1 *are mainly expressed in BNC, as previously reported [29,30,39-43]. *SULT1E1 *was also expressed in BNC (Fig. 4).

### QPCR analysis of transcription factor AP-2 family

**Figure 5:**
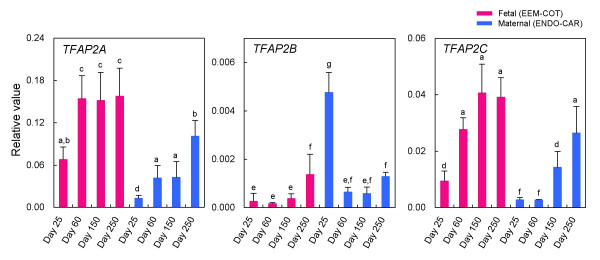
**QPCR analysis of *TFAP2A*, *TFAP2B*, and *TFAP2C *mRNA at each stage of bovine tissue (ENDO, CAR, EEM, COT)**. The gene expression on Days 25 ENDO (n = 3), 60 CAR (n = 4), 150 CAR (n = 3), 250 CAR (n = 4), 25 EEM (n = 3), 60 CAR (n = 4), 150 CAR (n = 3), and 250 CAR (n = 4) are shown. The expression of these genes was normalized to the expression of *GAPDH *measured in the same RNA preparation. The pink bar shows gene expression on fetal side (EEM to COT) by QPCR. The blue bar shows gene expression on maternal side (ENDO to CAR) by QPCR. Values are means ± SEM. Values with different letters are significantly different (*P *< 0.05).

The QPCR results are presented in Fig. 5. In EEM-COT, expression of *TFAP2A *increased from Day 25 to Day 60 and maintained a constant level up to the late stage of pregnancy. In ENDO-CAR, expression of this gene increased as gestation progressed, but the intensity in EEM-COT was higher than in ENDO-CAR at all stages. *TFAP2B *was expressed throughout gestation in both ENDO-CAR and EEM-COT. *TFAP2C *expression was low in ENDO-CAR and EEM-COT, but increased late in pregnancy. *TFAP2A *was more highly expressed than *TFAP2B *or *TFAP2C*.

### Localization of AP-2 family mRNA by in situ hybridization

**Figure 6:**
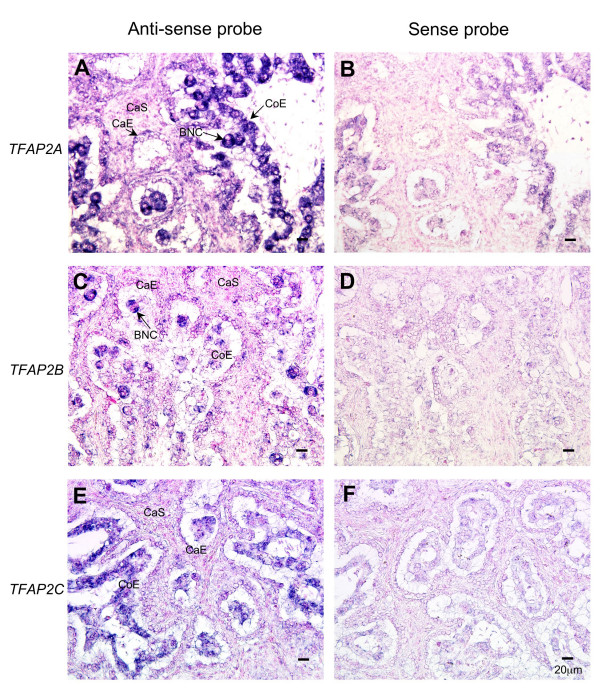
**Localization of *TFAP2A*, *TFAP2B*, and *TFAP2C *mRNA in the bovine placentome on Day 56 of gestation**. *TFAP2A *(A, B), *TFAP2B *(C, D) and *TFAP2C *(E, F) mRNA were detected by *in situ *hybridization. (A, C, E) DIG-labeled anti-sense cRNA probes were used. (B, D, F) DIG-labeled sense cRNA probes were used. Seven-micrometer sections of bovine placentome were hybridized with each probe. Scale bar = 20 μm. CaE: caruncular epithelium. CaS: caruncular stroma. CoE: cotyledonary epithelium. BNC: binucleate cell.

The cells expressing the AP-2 family were identified by *in situ *hybridization in the bovine placentome on Day 60 of gestation (Fig. 6). DIG-labeled *TFAP2A, TFAP2B *and *TFAP2C *anti-sense RNA probes specifically detected the mRNA transcripts in the placentome. *TFAP2A *appeared mainly in the cotyledonary villous epithelium (Fig. 6A). The principal expressing cells were in the cotyledonary villous epithelium, including the BNC. *TFAP2B *was specifically expressed in the BNC of cotyledonary villi (Fig. 6C). *TFAP2C *was specifically expressed in trophoblast mononucleate cells of the cotyledonary villi (Fig. 6E). No significant signals for any gene were detected with sense probes (Figs. 6B, D and 6F).

## Discussion

Placental and trophoblast gene expression profiles depend on the cells and tissues, as well as the period of gestation. Diverse expression profiles have been reported and spatially and temporally different expressions have been observed in bovine placentomes [4,5]. However, global gene profiles have not been available for the whole of gestation in bovine placenta. In previous studies, specific expression of genes in trophoblast cells, such as *CSH1*, *PRPs *and *PAGs *in BNC, has been noted because of marked changes in expression level [29,30,39-44]. In the present study, placentomal gene expression profiles during gestation were analyzed for their intensities and patterns. The factors proactive in regulating gene expression were also examined.

The most marked changes were found in genes related to trophoblast cells, as in previous reports [4,5]. In Cluster 2, the expression intensities of *CSH1*, *PAGs*, *PRPs*, *SULT1E1*, *TMSB10 *and others increased as gestation progressed. These genes are known to be among the crucial factors for implantation, placentation and the maintenance of gestation in cattle [30,40-42,44]. Various ECM-related genes expressed in the endometrium declined from the early to the late stages of pregnancy. Cluster 8 comprised ECM-related genes and proteinases and their inhibitors, such as *COL1A*2, *MGP*, *LAMB1*, *CLU*, *CST3 *and *CTSB*. Many of these may also play important roles in maintaining gestation and may be expressed specifically in the endometrium [4,45-47]. The genes in this cluster may mostly have specific roles in remodeling the endometrium throughout gestation, especially during implantation and placentation. In mice, both the cysteine proteinase CTSB and its inhibitor CST3 are expressed in the placenta; they are important in remodeling the ECM and forming the decidua [48]. These global gene expression data suggest that the genes grouped in the same cluster are related not only in showing a similar expression pattern, but also in having similar or opposite functions. For example, *COL1A2*, *MGP*, *LAMB1 *and *CLU *in cluster 8 have ECM-related functions. In contrast, *CTSB *and its inhibitor *CST3*, also expressed in cluster 8, have opposite functions. The global gene expression profiles in the present microarray study were confirmed by QPCR using selected genes (*ANXA1*, *CTDSP2*, *HSPA1A*, *HSPA8*, *MSX1 *and *SULT1E1*). The comparatively high reliability of the microarray data was confirmed by QPCR, as well as by previous studies [4-6]. These selected genes may have a central role in placental formation and function; it is suggested that ANXA1 inhibits inflammation in the human placenta [32]. CTDSP2 is the enzyme that dephosphorylates the C-terminal domain (CTD) of RNA polymerase II [33]. Unexpectedly, CTD phosphorylation was found not to be essential for RNA polymerase II-mediated transcription in mouse trophoblast giant cells [34]. Members of the HSPA family are expressed constantly throughout pregnancy until parturition in human placenta [35] in cytotrophoblast, syncytiotrophoblast, intermediate trophoblast and endothelial cells [36]. MSX1 is regulated by the leukemia inhibitory factor (LIF) or ovarian steroid hormones (estrogen and progesterone) in mouse endometrium specifically during the implantation period, and its expression decreased as implantation progressed [37]. SULT1E1 regulates active estrogen and is active in mid to late pregnancy [38].

A microarray can be used to collect the vast amount of data related to expression profiling and to monitor the expression levels of thousands of genes simultaneously. One of the goals of this work was to discover the transcription factors common to the regulation of gene expression during bovine reproduction [4,49]. We also examined whether the microarray results suggest a regulatory cascade of gene expression. Cluster 2 genes exhibited a characteristic expression pattern, increasing from the early to the late stage of pregnancy. This increase in gene expression suggests functional and morphological developments of the placentomes. Microarray analysis can be used as an exploratory tool for understanding the biological functions of placental cells.

A genome-wide analysis of a common transcription factor is one approach to utilizing microarray data effectively. *In silico *research involving the search for a common transcription factor by microarray data analysis has been reported [14-19]. We searched for a common regulatory element in the cluster in which the bovine trophoblast cell-specific genes appear using the TFBIND program [27].

The results indicated that an AP-2 binding site is common to the upstream (promoter) regions of the principal genes in cluster 2 (Fig. 3). It is known that the AP-2 family plays a role in the differentiation and proliferation of mouse, human and ovine trophoblast cells [50-54]. Embryos of TFAP2C-deficient mice die during the middle stage of development, suggesting that trophectodermal cells cannot proliferate in a TFAP2C-deficient mouse [51,52]. This research was mainly carried out using cultured human cells in which placental-specific genes such as *hCSH1*, human chorionic gonadotropin (*hCG*) and human corticotropin-releasing hormone (*hCRH*) are regulated by the AP-2 family [53,54]. There are some reports on the regulation of placenta-essential genes by the AP-2 family. Adenosine deaminase (ADA) is a purine metabolism enzyme that is enriched in trophoblast cells in the murine placenta. Studies on ADA-deficient mice have demonstrated that the absence of ADA from trophoblast cells is associated with perinatal lethality [55,56]. This gene is regulated by TFAP2C [51,57]. *hCG *is a placenta-specific gene in the human placenta and the expression of *hCGα *or *hCGβ *is also regulated by TFAP2A or TFAP2C [54,58-61]. *CSH1*, a gene with trophoblast cell-specific expression, is regulated by TFAP2A or TFAP2C in mouse, rat, human and sheep [62-65]. The binding site for AP-2 in *CSH1 *was identical to the site specified for ovine *CSH1 *[50,65]. The binding site in the bovine gene may again be similar because the upstream sequences in the orthologous genes resemble each other. An AP-2 binding site has also been reported in the promoter regions of mouse, rat and human *CSH1 *[54,63,64]. It is anticipated that *TMSB10 *and *SULT1E1*, which are expressed at high levels in the placenta, are similarly regulated by the AP-2 family, as determined by TFBIND analysis. In *PRP1*, the existence of an AP-2 binding site in the enhancer region (-1215 to -1204) has been reported, and the AP-2 family is predicted to bind at this site [66]. In our results, two AP-2 binding sites were newly confirmed by the TFBIND search at -74 to -63 and -44 to -33. AP-2 binding sites in the *PAG17 *promoter region were confirmed at three positions (Fig. 3). However, an AP-2 binding site in the *PAG1 *promoter region was confirmed only at -16 to -5 (Fig. 3) [67]. General transcription factors may occupy this site. AP-2 binding sites were also confirmed in the *PAG1 *enhancer region (-1026 to -1024) by a TFBIND search. It was possible to predict the integrated regulatory elements of gene clusters that specifically appeared in the trophoblast. Integrated transcriptional regulator analysis may be of value for investigating gene cascades on a genome-wide level.

The AP-2 family has splice variants. Three of these, *TFAP2A*, *TFAP2B *and *TFAP2C*, were examined in the present study to determine their expression intensities and locations. AP-2 genes were expressed mainly in the COT epithelium along with *CSH1, PRP1, PAG1 *and BCL2-related protein A1 (*BCL2A1*) [29,31,42,43]. However, localization of the expressing cells was dependent on the type of AP-2 variant. *TFAP2A *was confirmed in BNC and mononucleate cells. *TFAP2B *was confirmed in only BNC but its expression level was extremely low. *TFAP2C *was confirmed in trophoblast mononucleate cells an expression level mid-way between those of *TFAP2A *and *TFAP2B *(Fig. 6). These results suggest that this gene family may have different roles in the differentiation and proliferation of trophoblasts. In murine and human placenta, *TFAP2C *was found to be the most highly expressed of the AP-2 family; *TFAP2A *was detected in the trophoblast cell lineage (giant cells and cytotrophoblast cells), but *TFAP2B *was not completely confirmed [54,57]. *CSH1 *is specifically expressed in trophoblast giant cells in rodents, in syncytial trophoblast in humans and in BNC in ruminants [68]. Previous studies and the present study clearly suggest that the AP-2 family is a principal factor in regulating *CSH1 *functions in rodents and humans [54,62-65]. The present study also suggests that AP-2 regulates cytochrome P450-related genes for producing steroid hormones in bovine placental BNC [69,70]. Steroid hormone production and cytochrome P450 and SULT1E1 expression may also be linked by AP-2 regulation, because SULT1E1 sulfates estrone [38]. Recently, it was reported that an endogenous retrovirus regulates BNC differentiation in sheep [71,72]. The endogenous retrovirus may have an important role in developing viviparity, trophoblast cell migration and placental development, so the AP-2 family might be related to its expression. Because the appearance of BNC in the trophoblast cell lineage coincides with *CSH1* expression [40,73], the AP-2 family may play a crucial role in trophoblast cell differentiation, remodeling of the endometrium, implantation and maintenance of gestation in bovine placenta. However, the regulation of AP-2 gene expression and the role of AP-2 in bovine placenta remain unclear [74,75].

## Conclusion

Global gene expression analysis was performed on bovine placentomes using a microarray. The genes were partitioned into ten expression-profile clusters by *k*-means clustering. Some placental-specific genes such as *CSH1*, *PRP1 *and *PAG1 *were assigned to cluster 2. We searched for transcription factors common to the regulation of cluster 2 expression using *in silico *analysis. The results suggest that the AP-2 family includes such factors. The microarray and *in silico *analyses provided clues to the regulatory mechanism common to the crucial genes in bovine placenta. Expression of the AP-2 family in the placenta was quantified and localized. It was confirmed only in BNC or mononucleate cells. We deduced that the AP-2 family regulates genes that play a crucial role in placetogenesis. It is also suggested that the role differs for each gene in the AP-2 variants.

## Authors' contributions

KU participated in the design of the study, carried out most of the experiments and wrote the manuscript. TT participated in coordinating the design of the study. KU, TT, MH, KaKa, KeKi and KH collected the tissue samples. MH and KaKa were responsible for all animal care and the artificial insemination of cows. KU, HI and KeKi carried out the DNA microarray experiments and analysis. KH participated in coordinating the design of the study and helped to draft the manuscript. All authors read and approved the final manuscript.

## References

[B1] Wooding FBP, Flint AP, Lamming GE (1994). Placentation. Marshall's Physiology of Reproduction.

[B2] Wooding FBP, Morgan G, Monaghan S, Hamon M, Heap RB (1996). Functional specialization in the ruminant placenta: evidence for two populations of fetal binucleate cells of different selective synthetic capacity. Placenta.

[B3] Hashizume K, Ushizawa K, Patel OV, Kizaki K, Imai K, Yamada O, Nakano H, Takahashi T (2007). Gene expression and maintenance of pregnancy in bovine: roles of trophoblastic binucleate cell-specific molecules. Reprod Fertil Dev.

[B4] Ishiwata H, Katsuma S, Kizaki K, Patel OV, Nakano H, Takahashi T, Imai K, Hirasawa A, Shiojima S, Ikawa H, Suzuki Y, Tsujimoto G, Izaike Y, Todoroki J, Hashizume K (2003). Characterization of gene expression profiles in early bovine pregnancy using a custom cDNA microarray. Mol Reprod Dev.

[B5] Ushizawa K, Herath CB, Kaneyama K, Shiojima S, Hirasawa A, Takahashi T, Imai K, Ochiai K, Tokunaga T, Tsunoda Y, Tsujimoto G, Hashizume K (2004). cDNA microarray analysis of bovine embryo gene expression profiles during the pre-implantation period. Reprod Biol Endocrinol.

[B6] Ushizawa K, Takahashi T, Kaneyama K, Tokunaga T, Tsunoda Y, Hashizume K (2005). Gene expression profiles of bovine trophoblastic cell line (BT-1) analyzed by a custom cDNA microarray. J Reprod Dev.

[B7] Misirlioglu M, Page GP, Sagirkaya H, Kaya A, Parrish JJ, First NL, Memili E (2006). Dynamics of global transcriptome in bovine matured oocytes and preimplantation embryos. Proc Natl Acad Sci USA.

[B8] Degrelle SA, Campion E, Cabau C, Piumi F, Reinaud P, Richard C, Renard JP, Hue I (2005). Molecular evidence for a critical period in mural trophoblast development in bovine blastocysts. Dev Biol.

[B9] Cammas L, Reinaud P, Dubois O, Bordas N, Germain G, Charpigny G (2005). Identification of differentially regulated genes during elongation and early implantation in the ovine trophoblast using complementary DNA array screening. Biol Reprod.

[B10] Klein C, Bauersachs S, Ulbrich SE, Einspanier R, Meyer HH, Schmidt SE, Reichenbach HD, Vermehren M, Sinowatz F, Blum H, Wolf E (2006). Monozygotic twin model reveals novel embryo-induced transcriptome changes of bovine endometrium in the preattachment period. Biol Reprod.

[B11] Bauersachs S, Ulbrich SE, Gross K, Schmidt SE, Meyer HH, Wenigerkind H, Vermehren M, Sinowatz F, Blum H, Wolf E (2006). Embryo-induced transcriptome changes in bovine endometrium reveal species-specific and common molecular markers of uterine receptivity. Reproduction.

[B12] Wolf E, Arnold GJ, Bauersachs S, Beier HM, Blum H, Einspanier R, Frohlich T, Herrler A, Hiendleder S, Kolle S, Prelle K, Reichenbach HD, Stojkovic M, Wenigerkind H, Sinowatz F (2003). Embryo-maternal communication in bovine – strategies for deciphering a complex cross-talk. Reprod Domest Anim.

[B13] Brazma A, Jonassen I, Vilo J, Ukkonen E (1998). Predicting gene regulatory elements in silico on a genomic scale. Genome Res.

[B14] Gao F, Foat BC, Bussemaker HJ (2004). Defining transcriptional networks through integrative modeling of mRNA expression and transcription factor binding data. BMC Bioinformatics.

[B15] Kim SY, Kim Y (2006). Genome-wide prediction of transcriptional regulatory elements of human promoters using gene expression and promoter analysis data. BMC Bioinformatics.

[B16] Veerla S, Hoglund M (2006). Analysis of promoter regions of co-expressed genes identified by microarray analysis. BMC Bioinformatics.

[B17] Hvidsten TR, Wilczynski B, Kryshtafovych A, Tiuryn J, Komorowski J, Fidelis K (2005). Discovering regulatory binding-site modules using rule-based learning. Genome Res.

[B18] Werner T (2001). Cluster analysis and promoter modelling as bioinformatics tools for the identification of target genes from expression array data. Pharmacogenomics.

[B19] Kamalakaran S, Radhakrishnan SK, Beck WT (2005). Identification of estrogen-responsive genes using a genome-wide analysis of promoter elements for transcription factor binding sites. J Biol Chem.

[B20] Hashizume K, Ishiwata H, Kizaki K, Yamada O, Takahashi T, Imai K, Patel OV, Akagi S, Shimizu M, Takahashi S, Katsuma S, Shiojima S, Hirasawa A, Tsujimoto G, Todoroki J, Izaike Y (2002). Implantation and placental development in somatic cell clone recipient cows. Cloning Stem Cells.

[B21] Herath CB, Shiojima S, Ishiwata H, Katsuma S, Kadowaki T, Ushizawa K, Imai K, Takahashi T, Hirasawa A, Tsujimoto G, Hashizume K (2004). Pregnancy-associated changes in genome-wide gene expression profiles in the liver of cow throughout pregnancy. Biochem Biophys Res Commun.

[B22] MIAME. http://www.mged.org/Workgroups/MIAME/miame.html.

[B23] GEO. http://www.ncbi.nlm.nih.gov/projects/geo/.

[B24] Saeed AI, Sharov V, White J, Li J, Liang W, Bhagabati N, Braisted J, Klapa M, Currier T, Thiagarajan M, Sturn A, Snuffin M, Rezantsev A, Popov D, Ryltsov A, Kostukovich E, Borisovsky I, Liu Z, Vinsavich A, Trush V, Quackenbush J (2003). TM4: a free, open-source system for microarray data management and analysis. Biotechniques.

[B25] TM4. http://www.tm4.org/.

[B26] Map Viewer. http://www.ncbi.nlm.nih.gov/mapview/map_search.cgi?taxid=9913.

[B27] Tsunoda T, Takagi T (1999). Estimating Transcription Factor Bindability on DNA. Bioinfomatics.

[B28] TFBIND. http://tfbind.ims.u-tokyo.ac.jp.

[B29] Ushizawa K, Kaneyama K, Takahashi T, Tokunaga T, Tsunoda Y, Hashizume K (2005). Cloning and expression of a new member of prolactin-related protein in bovine placenta: bovine prolactin-related protein-VII. Biochem Biophys Res Commun.

[B30] Ushizawa K, Takahashi T, Hosoe M, Kaneyama K, Hashizume K (2005). Cloning and expression of two new prolactin-related proteins, prolactin-related protein-VIII and -IX in bovine placenta. Reprod Biol Endocrinol.

[B31] Ushizawa K, Takahashi T, Kaneyama K, Hosoe M, Hashizume K (2006). Cloning of the bovine antiapoptotic regulator, BCL2-related protein A1, and its expression in trophoblastic binucleate cells of bovine placenta. Biol Reprod.

[B32] Bennett P, Slater D, Berger L, Moor G (1994). The expression of phospholipase A2 and lipocortins (annexins) I, II and V in human fetal membranes and placenta in association with labour. Prostaglandins.

[B33] Dahmus ME (1996). Reversible phosphorylation of the C-terminal domain of RNA polymerase II. J Biol Chem.

[B34] Rossi DJ, Londesborough A, Korsisaari N, Pihlak A, Lehtonen E, Henkemeyer M, Makela TP (2001). Inability to enter S phase and defective RNA polymerase II CTD phosphorylation in mice lacking Mat1. EMBO J.

[B35] Divers MJ, Bulmer JN, Miller D, Lilford RJ (1995). Placental heat shock proteins: no immunohistochemical evidence for a differential stress response in preterm labour. Gynecol Obstet Invest.

[B36] Shah M, Stanek J, Handwerger S (1998). Differential localization of heat shock proteins 90, 70, 60 and 27 in human decidua and placenta during pregnancy. Histochem J.

[B37] Daikoku T, Song H, Guo Y, Riesewijk A, Mosselman S, Das SK, Dey SK (2004). Uterine Msx-1 and Wnt4 signaling becomes aberrant in mice with the loss of leukemia inhibitory factor or Hoxa-10: evidence for a novel cytokine-homeobox-Wnt signaling in implantation. Mol Endocrinol.

[B38] Hoffmann B, Falter K, Vielemeier A, Failing K, Schuler G (2001). Investigations on the activity of bovine placental oestrogen sulfotransferase and -sulfatase from midgestation to parturition. Exp Clin Endocrinol Diabetes.

[B39] Nakano H, Takahashi T, Imai K, Hashizume K (2001). Expression of placental lactogen and cytokeratin in bovine placental binucleate cells in culture. Cell Tissue Res.

[B40] Yamada O, Todoroki J, Kizaki K, Takahashi T, Imai K, Patel OV, Schuler LA, Hashizume K (2002). Expression of prolactin-related protein I at the fetomaternal interface during the implantation period in cows. Reproduction.

[B41] Kessler MA, Duello TM, Schuler LA (1991). Expression of prolactin-related hormones in the early bovine conceptus, and potential for paracrine effect on the endometrium. Endocrinology.

[B42] Patel OV, Yamada O, Kizaki K, Todoroki J, Takahashi T, Imai K, Schuler LA, Hashizume K (2004). Temporospatial expression of placental lactogen and prolactin-related protein-1 genes in the bovine placenta and uterus during pregnancy. Mol Reprod Dev.

[B43] Patel OV, Yamada O, Kizaki K, Takahashi T, Imai K, Hashizume K (2004). Quantitative analysis throughout pregnancy of placentomal and interplacentomal expression of pregnancy-associated glycoproteins-1 and -9 in the cow. Mol Reprod Dev.

[B44] Green JA, Xie S, Quan X, Bao B, Gan X, Mathialagan N, Beckers JF, Roberts RM (2000). Pregnancy-associated bovine and ovine glycoproteins exhibit spatially and temporally distinct expression patterns during pregnancy. Biol Reprod.

[B45] Brown TL, Moulton BC, Witte DP, Swertfeger DK, Harmony JA (1996). Apolipoprotein J/clusterin expression defines distinct stages of blastocyst implantation in the mouse uterus. Biol Reprod.

[B46] Bauersachs S, Ulbrich SE, Gross K, Schmidt SE, Meyer HH, Einspanier R, Wenigerkind H, Vermehren M, Blum H, Sinowatz F, Wolf E (2005). Gene expression profiling of bovine endometrium during the oestrous cycle: detection of molecular pathways involved in functional changes. J Mol Endocrinol.

[B47] Song G, Spencer TE, Bazer FW (2005). Cathepsins in the ovine uterus: regulation by pregnancy, progesterone, and interferon tau. Endocrinology.

[B48] Afonso S, Romagnano L, Babiarz B (1997). The expression and function of cystatin C and cathepsin B and cathepsin L during mouse embryo implantation and placentation. Development.

[B49] Dawson KA (2006). Nutrigenomics: feeding the genes for improved fertility. Anim Reprod Sci.

[B50] Liang R, Limesand SW, Anthony RV (1999). Structure and transcriptional regulation of the ovine placental lactogen gene. Eur J Biochem.

[B51] Werling U, Schorle H (2002). Transcription factor gene AP-2 gamma essential for early murine development. Mol Cell Biol.

[B52] Auman HJ, Nottoli T, Lakiza O, Winger Q, Donaldson S, Williams T (2002). Transcription factor AP-2gamma is essential in the extra-embryonic lineages for early postimplantation development. Development.

[B53] Cheng YH, Aronow BJ, Hossain S, Trapnell B, Kong S, Handwerger S (2004). Critical role for transcription factor AP-2alpha in human trophoblast differentiation. Physiol Genomics.

[B54] Richardson BD, Cheng YH, Langland RA, Handwerger S (2001). Differential expression of AP-2gamma and AP-2alpha during human trophoblast differentiation. Life Sci.

[B55] Wakamiya M, Blackburn MR, Jurecic R, McArthur MJ, Geske RS, Cartwright J, Mitani K, Vaishnav S, Belmont JW, Kellems RE, Finegold MJ, Montgomery CA, Bradley A, Caskey CT (1995). Disruption of the adenosine deaminase gene causes hepatocellular impairment and perinatal lethality in mice. Proc Natl Acad Sci USA.

[B56] Blackburn MR, Wakamiya M, Caskey CT, Kellems RE (1995). Tissue-specific rescue suggests that placental adenosine deaminase is important for fetal development in mice. J Biol Chem.

[B57] Shi D, Kellems RE (1998). Transcription factor AP-2gamma regulates murine adenosine deaminase gene expression during placental development. J Biol Chem.

[B58] Knofler M, Saleh L, Bauer S, Galos B, Rotheneder H, Husslein P, Helmer H (2004). Transcriptional regulation of the human chorionic gonadotropin beta gene during villous trophoblast differentiation. Endocrinology.

[B59] Knofler M, Saleh L, Bauer S, Vasicek R, Griesinger G, Strohmer H, Helmer H, Husslein P (2000). Promoter elements and transcription factors involved in differentiation-dependent human chorionic gonadotrophin-alpha messenger ribonucleic acid expression of term villous trophoblasts. Endocrinology.

[B60] LiCalsi C, Christophe S, Steger DJ, Buescher M, Fischer W, Mellon PL (2000). AP-2 family members regulate basal and cAMP-induced expression of human chorionic gonadotropin. Nucleic Acids Res.

[B61] Johnson W, Albanese C, Handwerger S, Williams T, Pestell RG, Jameson JL (1997). Regulation of the human chorionic gonadotropin alpha- and beta-subunit promoters by AP-2. J Biol Chem.

[B62] Richardson BD, Langland RA, Bachurski CJ, Richards RG, Kessler CA, Cheng YH, Handwerger S (2000). Activator protein-2 regulates human placental lactogen gene expression. Mol Cell Endocrinol.

[B63] El-Hashash AH, Esbrit P, Kimber SJ (2005). PTHrP promotes murine secondary trophoblast giant cell differentiation through induction of endocycle, upregulation of giant-cell-promoting transcription factors and suppression of other trophoblast cell types. Differentiation.

[B64] Ozturk A, Donald LJ, Li L, Duckworth HW, Duckworth ML (2006). Proteomic identification of AP2 gamma as a rat placental lactogen II trophoblast cell-specific enhancer binding protein. Endocrinology.

[B65] Limesand SW, Anthony RV (2001). Novel activator protein-2alpha splice-variants function as transactivators of the ovine placental lactogen gene. Eur J Biochem.

[B66] Ebbitt DM, Hurley WL, Kessler MA, McDonald DJ, Schuler LA (1989). Characterization of the gene corresponding to bovine placental prolactin-related cDNA I: evolutionary implications. DNA.

[B67] Xie S, Green J, Beckers JF, Roberts RM (1995). The gene encoding bovine pregnancy-associated glycoprotein-1, an inactive member of the aspartic proteinase family. Gene.

[B68] Soares MJ (2004). The prolactin and growth hormone families: pregnancy-specific hormones/cytokines at the maternal-fetal interface. Reprod Biol Endocrinol.

[B69] Yamada K, Harada N, Honda S, Takagi Y (1995). Regulation of placenta-specific expression of the aromatase cytochrome P-450 gene. Involvement of the trophoblast-specific element binding protein. J Biol Chem.

[B70] Sher N, Yivgi-Ohana N, Orly J (2007). Transcriptional Regulation of the P450scc Gene (CYP11A1) Revisited: Binding of GATA, CREB and AP-1 Proteins to a Distal Novel Cluster of cis-Regulatory Elements Potentiates AP-2 and SF-1 Dependent Gene Expression in the Rodent Placenta and Ovary. Mol Endocrinol.

[B71] Dunlap KA, Palmarini M, Varela M, Burghardt RC, Hayashi K, Farmer JL, Spencer TE (2006). Endogenous retroviruses regulate periimplantation placental growth and differentiation. Proc Natl Acad Sci USA.

[B72] Spencer TE, Johnson GA, Bazer FW, Burghardt RC, Palmarini M (2007). Pregnancy recognition and conceptus implantation in domestic ruminants: roles of progesterone, interferons and Endogenous retroviruses. Reprod Fertil Dev.

[B73] Nakano H, Shimada A, Imai K, Takahashi T, Hashizume K (2002). Association of Dolichos biflorus lectin binding with full differentiation of bovine trophoblast cells. Reproduction.

[B74] Li M, Kellems RE (2003). Sp1 and Sp3 Are important regulators of AP-2gamma gene transcription. Biol Reprod.

[B75] Jin Y, Norquay LD, Yang X, Gregoire S, Cattini PA (2004). Binding of AP-2 and ETS-domain family members is associated with enhancer activity in the hypersensitive site III region of the human growth hormone/chorionic somatomammotropin locus. Mol Endocrinol.

